# Deep learning and session-specific rapid recalibration for dynamic hand gesture recognition from EMG

**DOI:** 10.3389/fbioe.2022.1034672

**Published:** 2022-12-15

**Authors:** Maxim Karrenbach, Pornthep Preechayasomboon, Peter Sauer, David Boe, Eric Rombokas

**Affiliations:** ^1^ Department of Electrical and Computer Engineering, University of Washington, Seattle, WA, United States; ^2^ Department of Mechanical Engineering, University of Washington, Seattle, WA, United States; ^3^ Department of Statistics and Data Science, Carnegie Mellon University, Pittsburgh, PA, United States

**Keywords:** electromyography-EMG, human-computer interaction (HCI), dimensionality reduction, gesture recognition, encoder-decoder (ED) model, open hardware, hand tracking

## Abstract

We anticipate wide adoption of wrist and forearm electomyographic (EMG) interface devices worn daily by the same user. This presents unique challenges that are not yet well addressed in the EMG literature, such as adapting for session-specific differences while learning a longer-term model of the specific user. In this manuscript we present two contributions toward this goal. First, we present the MiSDIREKt (Multi-Session Dynamic Interaction Recordings of EMG and Kinematics) dataset acquired using a novel hardware design. A single participant performed four kinds of hand interaction tasks in virtual reality for 43 distinct sessions over 12 days, totaling 814 min. Second, we analyze this data using a non-linear encoder-decoder for dimensionality reduction in gesture classification. We find that an architecture which recalibrates with a small amount of single session data performs at an accuracy of 79.5% on that session, as opposed to architectures which learn solely from the single session (49.6%) or learn only from the training data (55.2%).

## 1 Introduction

The recognition and use of hand behavior for control is a technique with potential applications in a wide range of fields. Surgical teleoperation systems use force and pressure sensors to capture hand movements and relay control signals to remote robotic arms (Wen et al., 2013); (Wen et al., 2014). Myoelectric prostheses use residual electromyography (EMG) signals from the residual limb to control the degrees of freedom of the prosthesis Resnik et al. (2018). Applications in Extended Reality (XR), such as Virtual Reality (VR) and Augmented Reality (AR), generally use a form of hand tracking to capture gestures and perform recognition for control in human-computer interactions (HCI) Kong et al. (2021). EMG has thus far been focused mainly for prosthetic devices; however EMG can be potentially transformative for HCI for consumer XR. An EMG-based wearable might enable muscle-controlled interactions which could work around the limitations of optical hand tracking, like occlusion. To make this alternative control scheme possible, three main goals must be met: practical EMG signal acquisition hardware, a dataset to train a system or algorithm to make sense of the complexities in muscle signals for dynamic interaction recognition, and an understanding of how user-specific and session-specific factors can be accounted for in the recognition system. In this study, we present a proof of concept which achieves these objectives.

The first contribution of this publication is a publicly available dataset of simultaneous forearm sEMG and hand kinematic data. This dataset was collected on a single subject over the course of 2 weeks, resulting in 43 complete sessions totalling 814 min (13.5 h) of activity recorded. The data of each session consists of four main activities: a unlabelled object stacking task, an unlabelled sequence of quick poses, a labelled task of dynamic interaction gestures, and a labelled task of rapidly performed poses. To our knowledge, this dataset is unique in both the amount of sessions collected from a single subject, as well as the wide range of dynamic activities performed and collected. This dataset is called the MiSDIREKt (Multi-session Single-subject Dynamic Interaction Recordings of EMG and Kinematics) Dataset.

The second contribution is the proposal of a classification scheme which addresses the issue of inter-session differences. This architecture learns underlying session-invariant features by training on a wide range of sessions, but performs well on unseen sessions by using a quick recalibration. The architecture takes advantage of the ability of the BiLSTM layer to understand the temporal dependencies of EMG. This architecture also uses an encoder-decoder structure with a bottleneck layer to take advantage of non-linear dimensionality reduction for latent dimension representations.

## 2 Background

### 2.1 Acquisition of multichannel forearm EMG

EMG is an integral tool for investigating muscle activation, neuron activity, and human control, and is typically acquired according to the needs of the study. Intramuscular or needle EMG performs acquisition by injecting a sensor into the tissue. This provides more accurate localized readings with the downsides of an invasive procedure, such as mobility constraints, longer recovery time and discomfort. Surface EMG (sEMG) is a non-invasive alternative which uses electrodes to monitor electrical signals through skin contact, with the consequence of less accurate signals. Wet electrodes are applied to the skin with gel or paste and can provide high quality readings for a shorter period of time and are used for applications which require rapid movements. Alternatively, dry electrodes need no extra material and can provide quick and easy setup. However, they are prone to motion artifacts and are generally noisier, and so are used for more stationary applications Hinrichs et al. (2020). These sEMG electrodes may be wired, which can sample at high rate with low latency but the presence of wires impedes range of motion and introduces wire impedance. There exist also wireless options, which require a wireless communication protocol which can vary in sample rate and latency, but provide a better user experience due to the lack of connection to a host device.

Studies containing EMG acquisition have the option of creating their own device, which allows for customization of design parameters to be specific to the study Fang et al. (2013). This flexibility allows for studies like Cisnal et al. (2021), where an acquisition design also allows for real-time embedded control for a device. The other option is to use a commercial device. One of the most commonly used devices was the Myo Armband (Thalmic Labs, Google Inc) which was a wireless band consisting of 8 sEMG electrodes as well as an accelerometer, IMU, and gyroscope Matsugi et al. (2022). While commercial off-the-shelf products restrict parameters like sample rate and channel number, they do allow for easy use. A market-dominant device or hardware standard have not yet emerged, but we anticipate that soon there will be widely adopted devices in this space. Until then, there is a need for an open standard hardware specification.

### 2.2 Open datasets of EMG

Many publicly available datasets containing multichannel forearm sEMG recordings of hand pose and activities currently exist. The most extensive dataset is the NinaPro database Atzori et al. (2014), which is divided into 10 separate databases with varying modes of data, gestures, activities, and subjects. Many of these subsets are also motivated differently, resulting in some databases having a wider variation of tasks, such as object grasping tasks. NinaPro is the most widely known and used dataset, but there exist many more publicly available datasets concerning sEMG recordings of hand activities. These datasets differ in factors such as the EMG acquisition method, the number of subjects and sessions, the amount and nature of the hand activities, and coupled modes of recording. Table 1 shows a small sample of more publicly available datasets, though this is not an exhaustive list.

**TABLE 1:** List and details of some publicly available datasets. Each datasets contains value in a specific application, and the variation between datasets can be seen through the parameters listed.

Dataset	Subject/Session	Poses	EMG	Hand kinematics
UC2018 DualMyo Simão et al. (2018)	1 Subjects 5 session per	8 gestures 110 reps each	16 channel 200 Hz	N/A
CSL-HDemg Amma et al. (2015)	5 Subjects 5 session per	27 gestures x reps each	192 channel x Hz	N/A
KIN-MUS UJI Jarque-Bou et al. (2018)	22 Subjects 1 session per	26 gestures 1 rep each	7 channel x Hz	18 joint angles CyberGlove
Kaczmarek et al. (2019)	44 Subjects 2 sessions per	26 gestures 1 rep each	24 channel 5210 Hz	hand image Depth/RGB Camera
MyoArmband Dataset Côté-Allard et al. (2019)	40 Subjects 1 session per	7 gestures640 reps each	8 channel 200 Hz	N/A
Zhang et al. (2020)	13 Subjects 1 session per	21 gestures 390 reps each	8 channel 200 Hz	N/A

While some datasets include more dynamic movements, like object grasping Atzori et al. (2014), a majority of the publicly available datasets contain static isometric poses. While this style of recording is valuable for investigating the characteristics of static hand pose EMG, this lack of dynamic gestures can be detrimental in the pursuit of using EMG as an input for prosthetic or interface control. Training with this type of data can lead to inflated accuracies in performance evaluation because static isometric poses are easier to classify than dynamic poses. However, the algorithm trained on static poses may not be particularly useful because realistically, the user will make fluid and rapid dynamic movements in gesture interactions and must not be constrained to slow and discrete static poses. Additionally, training with carefully curated samples of isometric poses could lead to algorithms that do not perform well in a noisy environment and could degrade further with a user who does not perform the gestures in the exact same manner as the data collected.

### 2.3 Estimation of hand pose and discrete gestures from EMG

#### 2.3.1 EMG signal processing

EMG signals are generally noisy and a significant amount of preprocessing is usually applied to extract a useful signal. Basic preprocessing consists of using bandpass filters to remove unwanted signals, such as n oise from motion artifacts or power line interference Luca et al. (2010). Many applications perform further signal processing, known as feature extraction, to further transform the signal. The most common types of feature extraction involve time domain calculations (Root-Mean-Square, Variance, Mean Absolute Value, etc.) on the signal amplitudes and transformations in the frequency domain (Autoregressive Coefficients, Frequency Mean, etc) on the signal’s power spectral density Spiewak (2018).

Dimensionality reduction techniques seek to facilitate recognition by intelligently projecting the EMG signal to a lower number of dimensions. This approach depends not only on the number of channels or features, but also the width of the window used. These techniques typically project the data onto predefined basis functions with certain desirable properties. Linear Discriminant Analysis (LDA) and Principal Component Analysis (PCA) find feature subspaces by maximizing separability of groups and capturing maximum variation directions, respectively. Empirical mode decomposition Aziz et al. (2019) and related approaches learn representations for compression, building basis functions that best represent the data using the data itself. However, these have constrained a particular form for those representations that limits their expressiveness Schmid (2010). The appeal of neural network approaches are that they are flexible and configurable in many ways to capture and represent features of the data in a learned way. The technique of encoder-decoder learning provides a learned transformation by training an architecture to reduce the input down to few dimensions (encoding), and then to reconstruct the input from these dimensions (decoding). The resulting architecture can compress and reconstruct EMG signals Dinashi et al. (2022) without a significant loss of classification accuracy. Because of this effect, encoder-decoder networks have begun to be successfully used for EMG gesture recognition applications Ibrahim and Al-Jumaily (2018) as well as for creating control signals for prosthetic applications Vujaklija et al. (2018).

#### 2.3.2 Recognition of discrete gestures

Recently, machine learning approaches have been used to recognize static poses and gestures by classifying EMG signals. Early studies in this field used more classical approaches of kNN and Bayesian classifiers Kim et al. (2008); Chen et al. (2007). More recently, deep learning architectures have achieved unprecedented successes by taking advantage of larger datasets and highly expressive and specialized classifier architectures. Architectures like deep neural networks and multi-layer perceptron classifiers have shown promise in gesture recognition Fajardo et al. (2021), but the most popular type of machine learning architecture in this task is the Convolutional Neural Network (CNN). Many studies choose to use the CNN in conjunction with the feature extraction techniques which create image representations of the signal, like Fourier Transforms or spectrograms. Windowing can create images in time for the CNN architecture inputs as well.

Due to the dynamic temporal dependencies of EMG, architectures more suited to time-series data have also been investigated, such as Recurrent Neural Networks (RNN), Gated Recurrent Units (GRU) or Long Short Term Memory (LSTM). Simão et al. (2019) compared the performance of three time-series architectures (RNN, GRU, LSTM) on a gesture recognition classification on a several datasets of 8 gestures. The authors found that the three architectures all tested above 95% on their own dataset and above 91% on a NinaPro DB5 subset. Jabbari et al. (2020) explored the ability of a stacked LSTM architecture to classify different gestures with different forces levels. This study used a dataset consisting of six different grip poses at three levels of force each (low, medium, high), collected over nine subjects with amputation. Results showed that the architecture performed at an accuracy of 91%, establishing the architecture’s ability to be able to distinguish not only grip poses but the forces behind these grips. Zhang et al. (2020) used a GRU architecture on raw EMG windows to classify a dataset of 21 gestures of both finger and wrist gestures to an accuracy of 90%. These results suggest that the time-series architectures are capable of recognizing gestures at a high accuracy comparable to the conventional CNN architecture.

Comparisons between results of studies can be difficult due to dataset differences, like the number and type of gestures, number of subjects and sessions, and any class imbalances. Differences in training scheme, particularly the training-validation-test split, as well as factors in the data collection, such as electrode placement or surface contact, will also introduce discrepancies between studies.

#### 2.3.3 Inter-session classification

Collected EMG data can differ drastically between subjects based on individual anatomy. The data may even differ between sessions of the same individual depending on electrode placement, environmental differences or presence of noise. Performance in gesture recognition from EMG signals suffers on unseen subjects, causing applications like prosthetic controllers to be highly subject-specific, requiring much training for the subject.

Domain adaptation is a technique for using a large, labeled source domain of data to train, and transferring representations to a unlabeled target domain which shares some similarity in labels to the source. This approach has largely been used for vision and image problems Xie et al. (2022); Li et al. (2021). Some approaches to this problem explore the varying domains between subjects or sessions and use architecture choices or adaptation to realize a subject-specific architecture from a source architecture trained on multiple differing subjects or sessions. Côté-Allard et al. (2019) proposed a Transfer Learning (TL) architecture in which a source network is trained with the data from all subjects with a subset of data. Then, a target network is trained for each subject specifically while the weights and most parameters of the source network are fixed. These architectures are merged, through layer-by-layer element-wise summation, during the training of the target network on the unseen subjects, with rates of success comparable to aforementioned studies. Du et al. (2017) introduced a deep domain adaptation approach, using an algorithm which learned a source domain distribution from the training data separately from a target domain distribution from the test data. This approach used adaptive batch normalization on unlabeled recalibration data and fine-tuning with labeled recalibration data when possible, showing significant improvement when implementing the adaptation.

Other approaches use the idea of retraining or recalibration by updating weights within the architecture based on previous predictions to improve subject-specific performance. Zhai et al. (2017) proposed a retraining scheme consisting of correcting previous predictions motivated by the tendency of sEMG to drift during a session. This retraining provided an approach which required no new data, and found significant improvements. Zheng et al. (2022) proposed an adaptive K-Nearest Neighbor (kNN) architecture for user independent gesture recognition by attributing weight changes based on the contribution of each sample to the overall classification. The architecture allows for incorrectly classified samples to have a smaller weight upon the distribution in which it was misclassified and for correctly classified samples to have larger weights in the correct distribution. This approach found success comparable to the literature especially with those using datasets with smaller numbers of gestures. Another promising approach, used for intracortical brain computer interface signals Ma et al. (2022), is to align the distributions of latent representations from different sessions using generative models.

COAPT Resnik et al. (2018) is an example of a commercially-available EMG interface that provides the user with the opportunity to recalibrate. A particular sequence of motions, the same every time, is performed. The EMG signals used to perform the sequence are used to replace, or update, the interface parameters. This is useful because the ground truth of the desired motions does not need to be sensed, but is instead prearranged. In this manuscript, we take inspiration from this idea in the form of the Hand Dance, described in more detail below, which is a representative sequence of motions that the user could repeat whenever recalibration is required.

## 3 Methods

### 3.1 Hardware

#### 3.1.1 Virtual reality

Our data collection virtual reality application was developed in Unity (Unity Technologies) and designed to run as a standalone application on the Meta Quest 2 (Reality Labs, Meta) virtual reality headset. We use the headset’s built-in hand-tracking to collect the hand’s kinematic pose and present physically-simulated hands to the user Preechayasomboon and Rombokas (2021). The high frame rate hand-tracking mode was used (60 Hz), but our hand kinematic data is sampled at the headset’s display frame rate with an average of 72 Hz.

#### 3.1.2 EMG acquisition hardware

**FIGURE 1:**
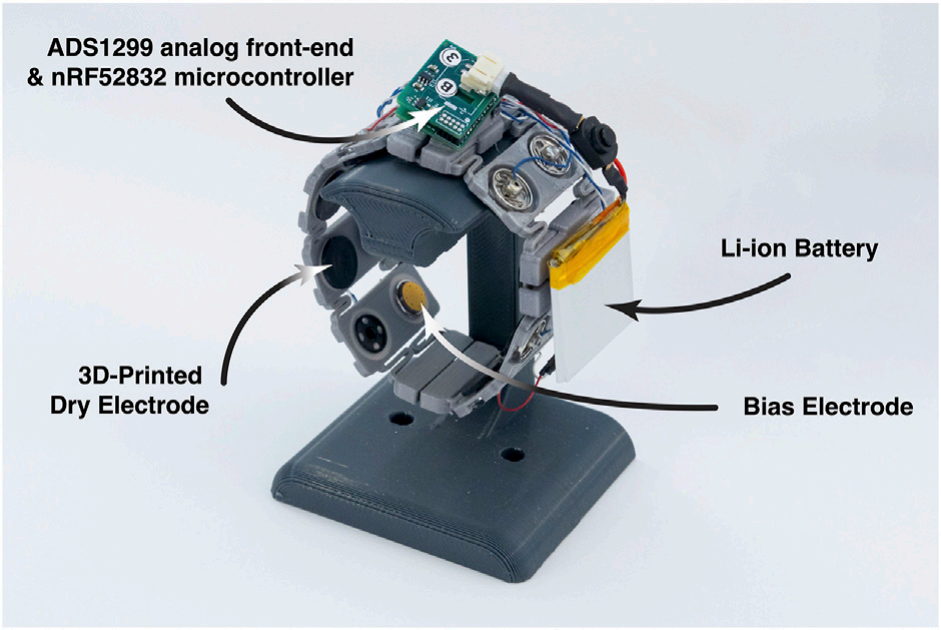
Apparatus used to collect the EMG data. It is a wireless 8-channel surface EMG device for data collection and input for AR and VR. The compact embedded system uses commercially-available hardware and the dry electrodes may be easily 3D printed.

For data collection, we fabricated a custom wireless 8-channel surface EMG data acquisition armband, as shown in Figure 1. The armband’s electronics is a derivative of the OpenBCI Cyton (OpenBCI), miniaturized to a 19 mm by 22 mm footprint. We use an ADS1299 (Texas Instruments) as our analog front-end (AFE) for signal amplification and analog-to-digital conversion, and an nRF52832 (Nordic Semiconductor) microcontroller for wireless data streaming. Our armband’s system can stream 8 channels of 24-bit EMG readings at 1,000 Hz wirelessly to USB dongle that forwards the readings to a host PC, smartphone or virtual reality headset.

Our armband’s electrodes are dry electrodes 3D-printed from electrically conductive carbon-black infused thermoplastic polyurethane (TPU), PI-ETPU 95–250 Carbon Black (Palmiga Innovation). The electrodes are circular in shape with a diameter of 14 mm and have brass snap fasteners. We 3D-printed a flexible armband using Shore 98A TPU to house pairs of electrodes circumferentially, and connect the electrodes using snap fasteners wired to our wireless EMG system. A more thorough description of hardware design and characterization of the electrodes, including dependence on mechanical grounding with the skin, may be found in Prechayasomboon and Rombokas (2023).

### 3.2 Data collection

The dataset of simultaneous EMG and hand kinematic data was collected over the span of 12 days. The subject was a left-handed male with no musculoskeletal impairments. All experimental procedures were approved by the Institutional Review Board of the University of Washington (STUDY00011627). The subject performed between one and four sessions per day, wherein one session consisted of four unique tasks: Hand Dance, Stacking, Gestures, Dynamic Interactions. The subject always began and ended with the Hand Dance task, which was designed as a quick series of discrete gestures to warm up before and cool down after the other three main tasks. After the initial Hand Dance task, the subject was presented with the three other tasks, which are described more in detail down below, in a pseudo-random order. While the Stacking and Dynamic Interaction tasks were performed only once, the Gestures task was performed twice, though never consecutively. Finally, the subject would perform the Hand Dance task again to complete the trial.

#### 3.2.1 Stacking task

**FIGURE 2:**
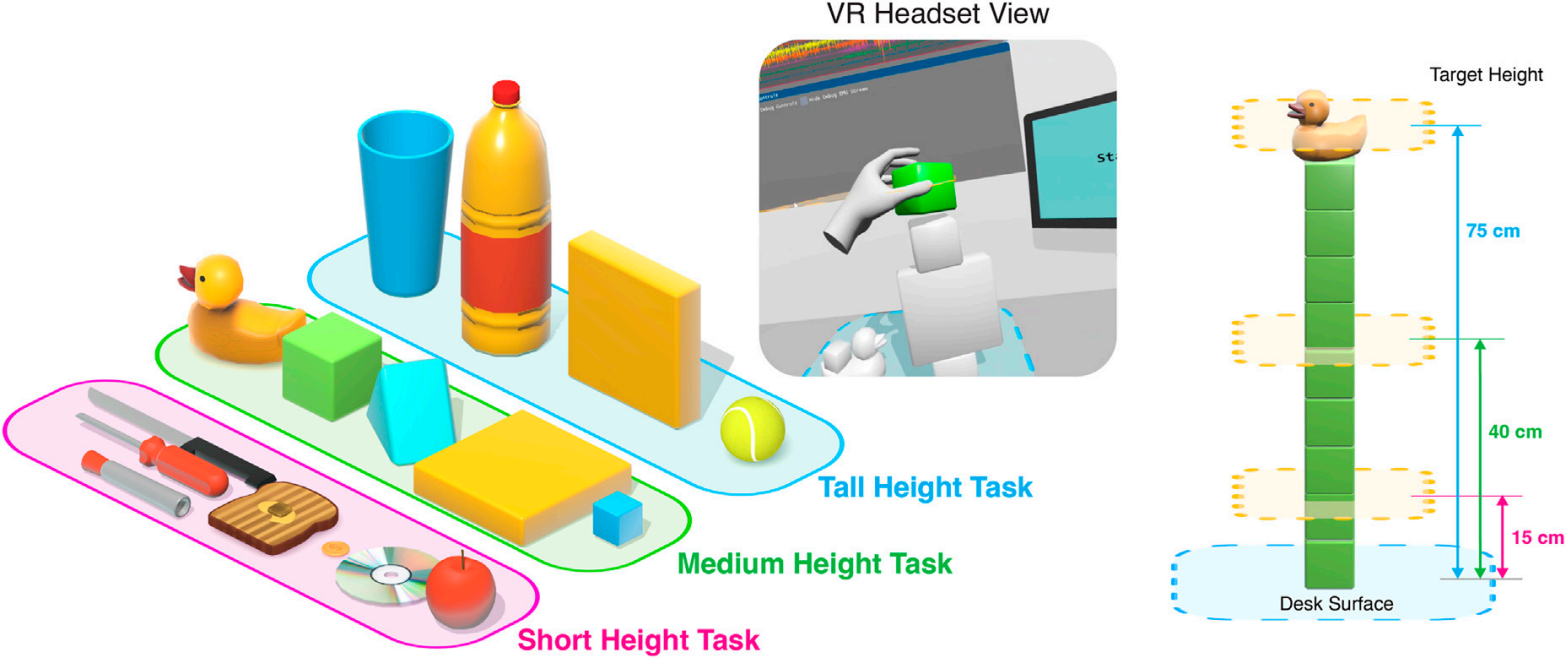
The description of the stacking task. Shown are the objects used for each for each of the target heights during the task, as well as the view of the user. The various target heights are described numerically.

The goal of the stacking task was to stack a series of objects past a specific height. The stacking objects task was performed for a total of nine repetitions, with three times at three different heights: a “tall” height, “medium” height, and “short” height. The objects, described in Figure 2, were chosen such that the user would use a certain grip, and are categorized either as primitive objects or function-based objects. The primitive objects were chosen to create general power and precision grasps described from grasp documentation studies Feix et al. (2016); Cutkosky (1989) while function-based objects were more complex and created more specific grips intended for task actions, described in a study for grasp patterns Kamakura et al. (1980). The dynamic, varying nature of grasping, manipulating, and placing objects is valuable to represent natural interactions with objects and to provide unsupervised data from unstructured movements, which were inspired by real-life everyday tasks Atzori et al. (2016).

#### 3.2.2 Gestures

**FIGURE 3:**
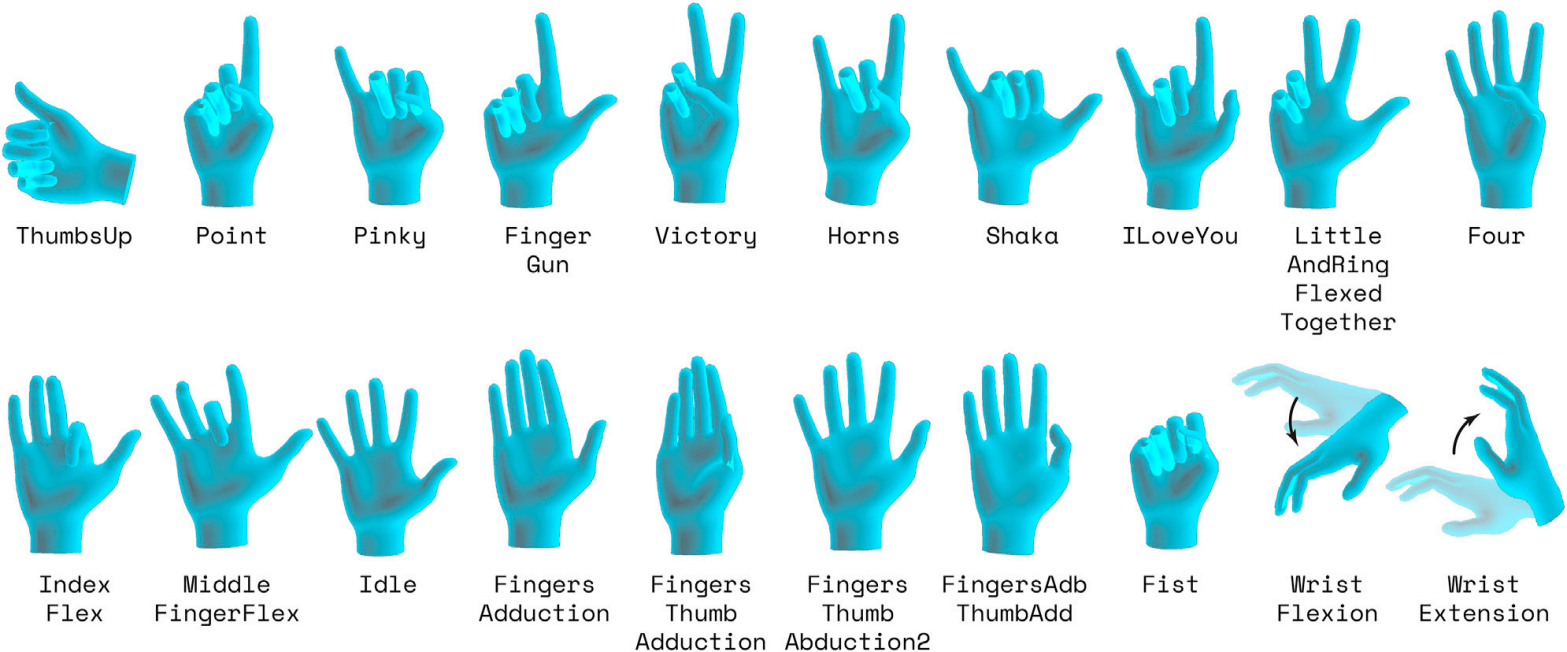
The 20 gestures for the In-Air Gestures Task, with the Idle pose being a representation of the general shape of the hand when subject rested. Note that for the visualization, poses may not be in the orientation as they were when actually performed. Subject would transition between the pose and an neutral pose for each pose.

The In-Air Hand Gestures consisted of a series of 20 hand gestures, shown in Figure 3, given in random order for the subject to perform. For each in-air hand gesture, a subject would observe a hand gesture in the virtual environment. Once prompted to start, the subject had 5 seconds to complete as many repetitions of that gesture as they pleased. The subject was not instructed to complete as many repetitions of the gesture as possible, but was encouraged to complete multiple iterations of the gesture. In between each distinct gesture, there was a small, periodic idle phase. This idle pose allows fingers to relax and minimizes EMG activity between gestures. The chosen poses were based on previous studies on EMG robotic and prosthesis control Atzori et al. (2016); Sebelius et al. (2005); Crawford et al. (2005). These movements have minimal self-contact, contain the most kinematic variability, and are valuable for constructing a latent kinematics manifold. These movements were also explicitly used in a gesture classification study Crawford et al. (2005) which used EMG data as an aim to control a prosthetic arm.

#### 3.2.3 Dynamic Interactions

**FIGURE 4:**
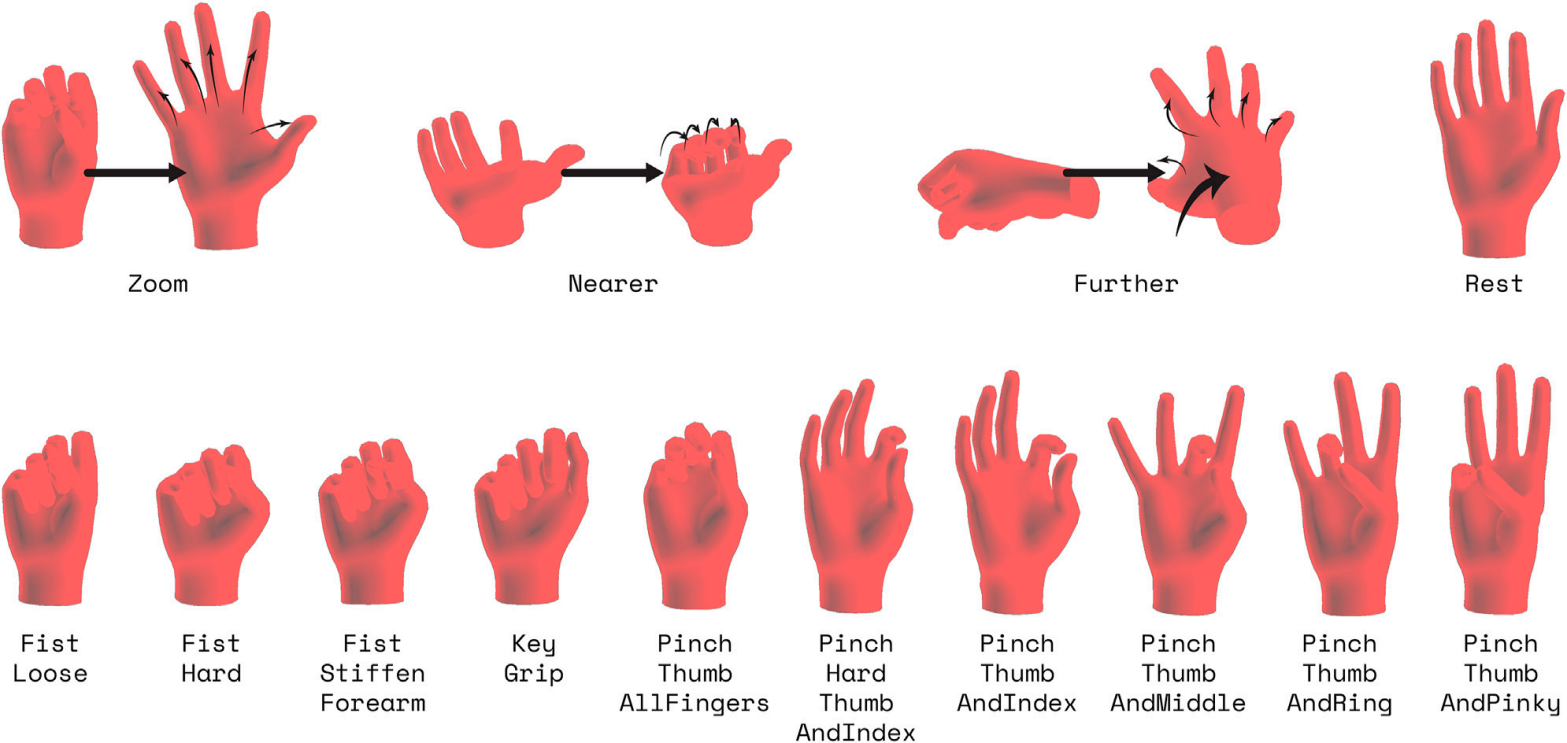
Visualizations of the gestures for the Interaction Gestures Task. Note that for the visualization, poses may not be in the orientation as they were when actually performed. For these poses, the user would start in a neutral pose with similar wrist orientations, then perform the pose.

In the Interaction Gestures task, the subject performed a series of 14 dynamic hand gestures, shown in Figure 4 given in random order. The subject was first cued to prepare for the gesture for a second, then the subject was prompted to perform the gesture and hold it for a second, after which the subject returned to a neutral pose. This neutral pose was self-selected by the subject when performing it, and differs from the specific “Rest” gesture included in this task. As opposed to the In-Air Hand Gestures task, subjects performed and held the gesture at a cued, specified time instead of performing as many repetitions as they pleased. For this task, the subject performed 10 repetitions of each gesture. The movements were based on modern mobile-device smartphone gestures, as well as actions we would believe to be useful in a 3D, virtual environment for XR applications.

### 3.3 Classification

To analyze a subset of the data and how to utilize the sessions of data, we consider a classification task on the dynamic interaction gestures described in Section 3.2.3 and shown in Figure 4. Although in the figure they appear to be specific poses, they are more like gestures. They are dynamic and more transitory than typical isometric poses from previous literature. These are poses appropriate for interacting with a user interface, and are somewhat similar in timing and execution from session to session. This means that, compared to the other parts of the dataset, session specific differences are more influenced by EMG acquisition differences than behavioral variability.

#### 3.3.1 Network architecture

We explore the time-series capability of the BiLSTM layers, as recurrent layers like the LSTM layers are able to identify and extract important information from time series data. With the complicated nature of temporally dynamic EMG, BiLSTM units are used in this architecture due to their ability to outperform LSTM units, albeit with slower convergence Siami-Namini et al. (2019). We also employ the encoder-decoder architecture as a form of dimensionality reduction, as it forces the high-dimensional input data to be represented in a smaller number of latent dimensions. The architecture used in this paper takes inspiration from the autoencoder architecture, which contains a bottleneck layer Hinton and Salakhutdinov (2006) through which the latent representation is created. This latent representation is not inherently interpretable, but can provide information like clusters or separability through visualization and analysis. This architecture is not a true autoencoder, since autoencoders aim for reconstruction of the original signal, while this architecture aims to perform classification. However, apart from the output layer, the symmetric form Hinton and Salakhutdinov (2006) of the autoencoder is used, where the decoder consists of a mirror network to the encoder. This encoder has previously been shown proven to extract useful latent representations for lower-limb activities Boe et al. (2021) and hand movements Portnova-Fahreeva et al. (2020, 2022).

**FIGURE 5:**
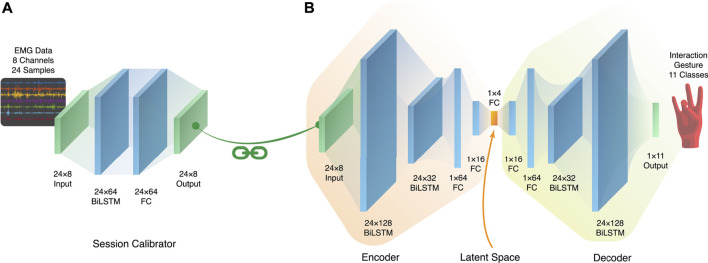
**(A)** The session-calibrator architecture, which preserves the shape of the EMG window. **(B)** The main encoder-decoder LSTM architecture used for training. The symmetry and bottleneck mimic that of a standard autoencoder.

Hyperparameter optimization on this architecture was performed on a subset of five sessions from the dataset, where each session had a training/validation split of 60%/40%. These sessions were the first sessions of the day for the first 5 days, because these sessions would represent situations where the user had the least amount of bias or familiarity, as the subject would not be influenced or possibly fatigued from a previous session. This approach found that two layers of BiLSTM and two fully connected layers, for each portion of the architecture, provided the best performance. To provide resilience against overfitting, dropout layers were inserted between each of the layers with a rate of 0.25. For the final training, the architecture used the Adam optimizer with a learning rate of 0.01, batch size of 128, and Early Stopping to further mitigate overfitting. For each session during training, an average of 20 epochs was used; however this number changed based on the Early Stopping and the particular session. Figure 5 shows the details of the shapes and types of each of the layers.

#### 3.3.2 Data processing

All of the EMG data in this dataset was processed in the same way, starting by scaling the data to millivolts. Next, a set of filters was applied, consisting of a bandpass filter from 60–500 Hz to remove motion artifacts, as well as two notch filters at 60 and 120 Hz, to remove power line interference. For this classification task specifically, the EMG data was also downsampled from 1,000 Hz to 72 Hz. A low pass filter was first applied to avoid alias artifacts; the filtered data was then downsampled by extracting the EMG signal at the time instances closest to those of the kinematic frames. This was done to match the sampling rate of the hand kinematics and to ensure that the labels had a corresponding sample in each data mode.

For a given session, the relatively static poses were extracted from the interaction gesture subset of data. The following gestures were removed from this task: Nearer, Further, Zoom. Each allowed pose is held statically for one second, which results in 60 samples. Each session has 10 instances of each pose resulting in around 500–600 samples of each pose per session, due to tracking errors and edge trimming. Each sample corresponds with a 8 × 24 window of the processed EMG data, which corresponds to a time window of 330 m.

**FIGURE 6:**
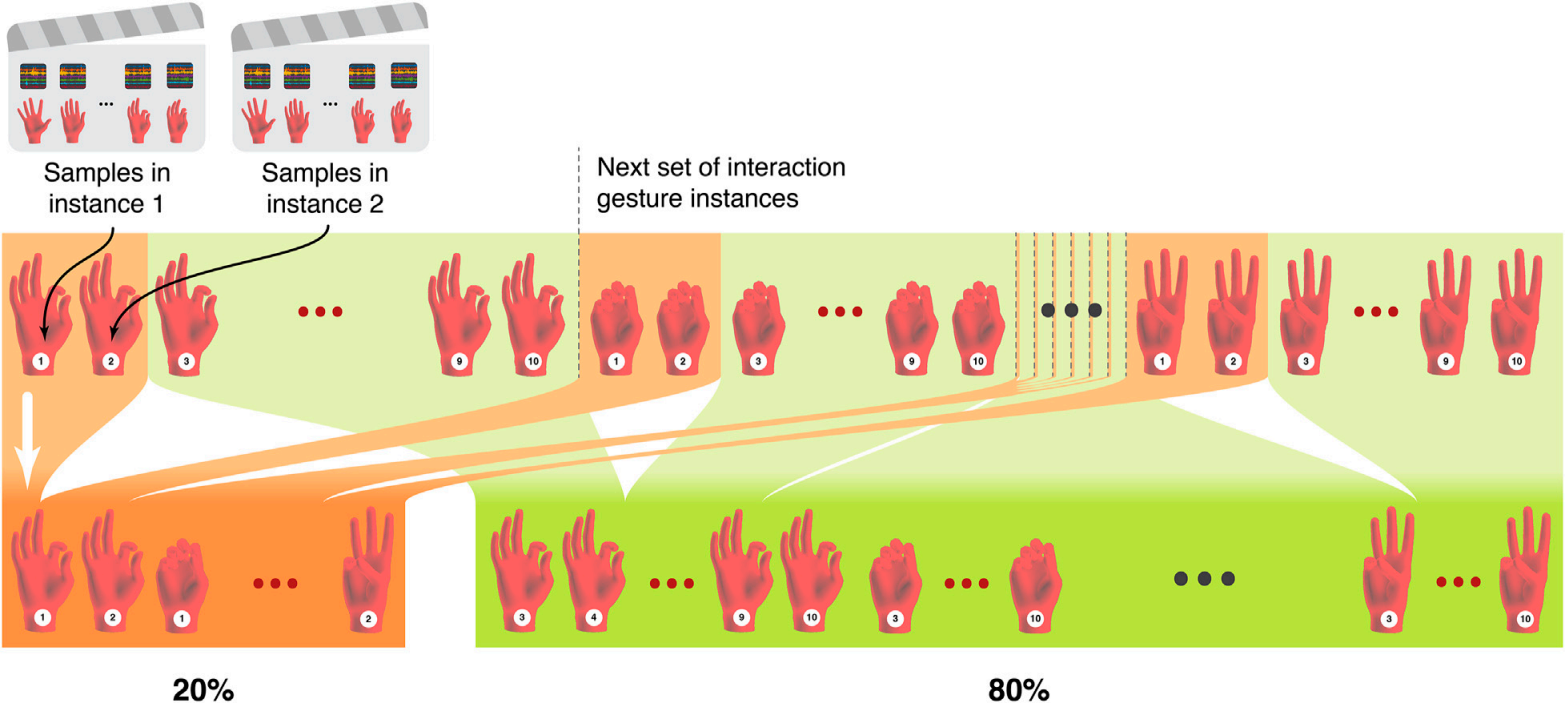
An example of the split of a specific session into independent percentages by separating based on particular pose examples. This ensures that splits include equal examples of poses, and are not simply randomly selected.

Each of the training schemes requires a train/validation/test split. We do not take a random percentage of the data for each split because this may cause issues with independence in the test set. This is because windowing creates overlap when the stride length is smaller than the window length, so two consecutive samples will contain a portion of identical data. Instead of random splits, we split along lines of each pose instance, shown in Figure 6. For example, if we require a split of 80% and 20%, we will take the first eight pose instances of each pose, which eliminates any possibility of having any samples from these eight instances in the remaining 20%, the remaining two pose instances of each pose. Because of the windowing overlap, using random splits would most likely introduce data leakage between the training and testing sets, as two consecutive samples which contain portions of identical data could be split into different sets. Using our proposed method, data leakage becomes less of a concern, since there is an amount of idle time between each instance that is larger than the time windowing parameter. This process allows our training, validation, and test sets to have independence from each other for proper validation and evaluation.

#### 3.3.3 Training and calibration

There are significant differences in the raw data collected between sessions, despite the data being collected from the same subject. To understand if every session is so distinct such that there is no shared information between sessions and if there is any value in this large amount of data, we propose to compare three different training schemes for classification:

• Single-Session Scheme
• Cumulative Scheme
• Recalibration Scheme

For the cumulative and retraining schemes, we use the first 38 sessions as a training set, and for each of the schemes, we use the last five sessions for the test set during evaluation.

##### 3.3.3.1 Single-session scheme

In applications, particularly in EMG-controlled prosthesis, a control algorithm can be created for the specific subject by training over a large amount of data from a single user. To imitate this, our first baseline training scheme (“Single-Session Training”) trains the architecture using the first 70% of pose data from one specific session, validates on the next 10% and tests the performance on the remaining 20% of the poses. This training uses the encoder-decoder architecture described in Figure 5B. This type of training scheme is represents the choice one might make for creating the most specialized algorithm for a particular session, but may not generalize well as it has the possibility of overfitting to the training data and learning the session’s irregularities.

##### 3.3.3.2 Cumulative Scheme

A draw of machine learning is that architectures can take advantage of large amounts of data to learn hidden general relationships. To understand how much underlying commonalities exist between sessions and how much impact that these session-independent relationships have on performance, our second baseline training scheme (“Cumulative Training”) trains the architecture using the training set, with each session being split as the first 80% of data for training and the last 20% for validation. The performance is tested on 100% of the unseen test set, as there is no session-specific training required. This training also uses the encoder-decoder architecture described in Figure 5B. This type of training scheme is common in many machine learning approaches to learn general trends in the data, but may also be unable to learn specific trends of particular sessions.

##### 3.3.3.3 Recalibration Scheme

**FIGURE 7:**
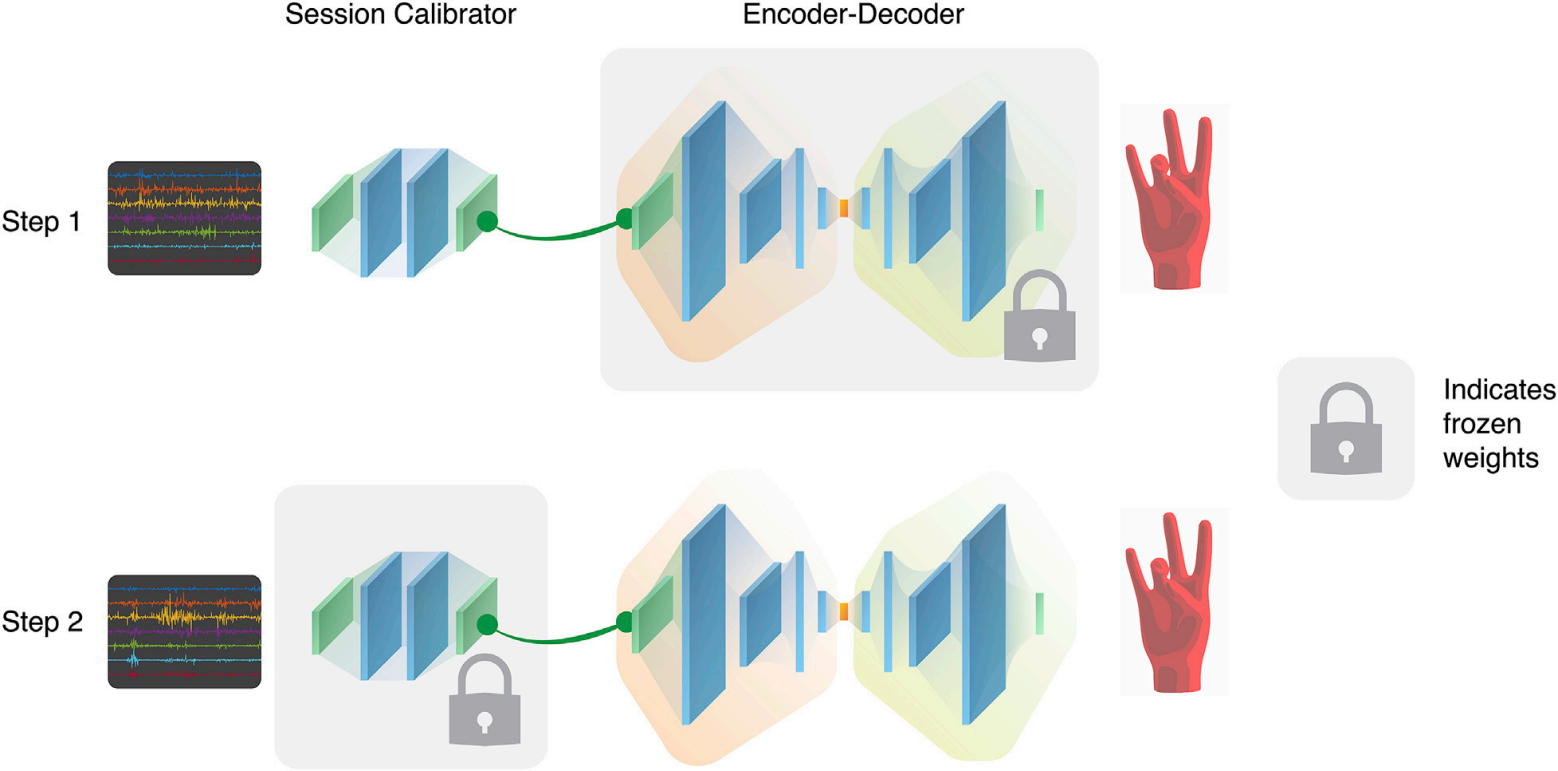
The proposed session-specific calibration scheme, which has two stages in both training and evaluation. Whenever a new session is introduced, a new session-calibrator architecture is created and trained while freezing the encoder-decoder architecture. Once the session-calibrator is trained, it is frozen, and transforms the inputs into a more session-independent format to be used as inputs to the encoder-decoder. The session-calibrator is frozen and the encoder-decoder is trained on the current session, with less session-specific bias.

We imagine that a high quality algorithm should be able to take advantage of large amounts of data as well as being generalizable to specific sessions. To accomplish this, we unite the previous two approaches for our third training scheme (“Recalibration Training”). Shown in Figure 7, a different training scheme is used to isolate the session-specific differences from the common relationships shared across all sessions. For each training session, the data is split into the first 80% for training and the remaining 20% for validation. Using this split, a session-calibrator architecture, shown in Figure 5A, is trained and validated while the weights and parameters of the encoder-decoder architecture, shown in Figure 5B, are frozen. Then, the encoder-decoder is unfrozen while the session-calibrator is frozen in the same way, and training is done again using same data split. By first recalibrating, the data from the specific session is transformed to not contain the irregularities of the session and so the encoder-decoder is not constantly updating itself with session-specific information. This allows for the encoder-decoder to extract the underlying similarities in gestures while the recalibrating learns to transform the data by mitigating the effect of session differences. To evaluate performance for each of the unseen sessions in the test set, a rapid re-training of the architecture is done first on a small “calibration portion” of session data, which is the first 20% of the data. The remaining 60% of the data from that session is still unseen to the user and is used for the evaluation.

## 4 Results

### 4.1 Classification performance

**FIGURE 8:**
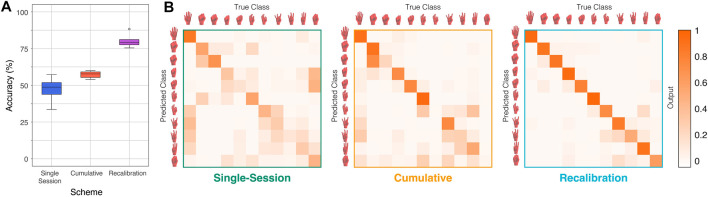
**(A)** The distribution of classification accuracies for each training scheme. **(B)** The confusion matrices corresponding to each training scheme. The improvement in performance is shown through the misclassification tendencies and the most misclassified poses.

The recalibration training scheme has the highest performance amongst the three training schemes at an average of 79.5%, while the single-session and cumulative training schemes have lower accuracies of 49.6% and 55.2%. These accuracies reported, shown in more detail in Figure 8A, are an average taken over the five test sessions that are unseen to the architecture during training. For the two training schemes that involve some form of training (single-session and recalibration), 5-fold cross validation was performed twice for each test session to ensure robustness of the results. Each fold is split based on instances and not random splitting to ensure no data leakage potential between the training and testing sets. The difference between the data split of each fold lies in which instances of each pose are selected for training and testing. For performance analysis, the average accuracies of the folds are used for these schemes. For the cumulative training, training was performed on the same five test sessions.

The results show that gestures with similar kinematic movements are more often misclassified as each other. The confusion matrix for each of the training schemes, as shown in Figure 8B, reveals that gestures within a group, like the fist gestures or the finger dexterous movements are more often misclassified as each other. Furthermore, exertion of force seems to also be apparent as a source of misclassification, as the more forceful movements, like the clenched fist and hard pinch, are also sometimes misclassified with each other. In the most successful training scheme, the confusion matrix reveals that there are still some misclassifications but mainly between gestures of similar types and less often.

### 4.2 Latent dimension representation

**FIGURE 9:**
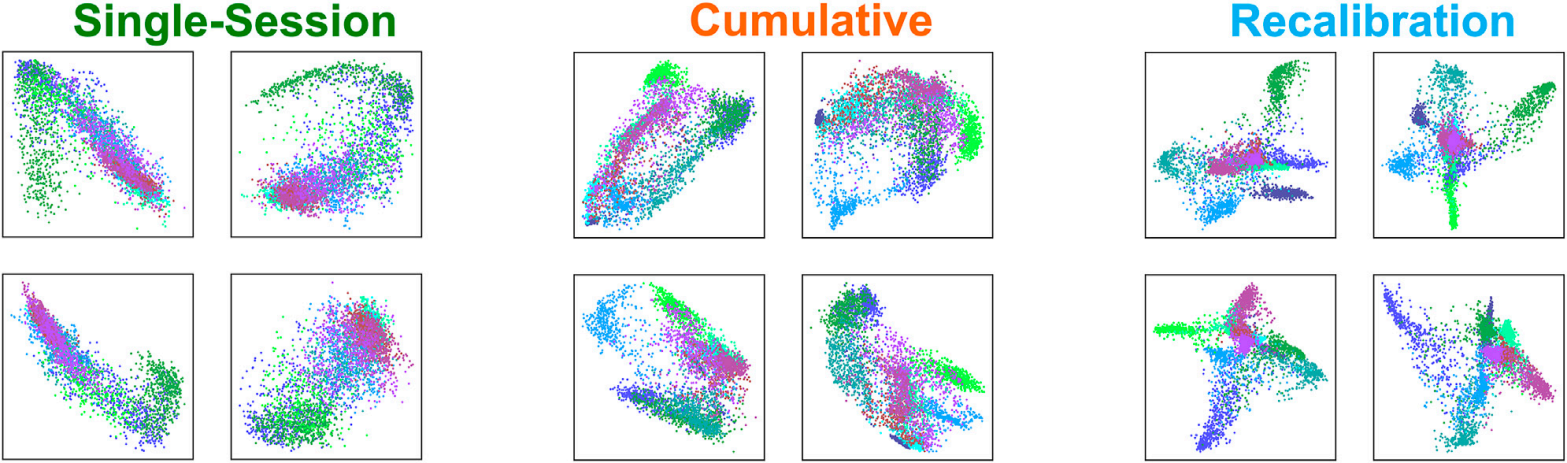
Each set of four panes represent an average latent dimension representation created from the bottleneck layer of the encoder-decoder for the single-session, cumulative, and recalibration schemes. As there are four dimensions or activations in the bottleneck, we display them plotted against each other for visualization. Each pose corresponds to a different color and separability is being shown between the various clusters of poses.

The latent representations of the sessions are shown in Figure 9, as the training progresses, the latent representations show improvement in the visual separability. The single-session scheme seems to show only a few main groupings with little variation within these groupings, while the cumulative scheme starts to show some differentiation in the latent representations. When the session calibrator architecture is trained, large clusters become much more defined and separable, and there is also focus around separating the classes within the larger clusters. The small pose clusters also show tighter distributions as opposed to the larger clouds seen in the other training schemes.

### 4.3 Multi-session benefit

**FIGURE 10:**
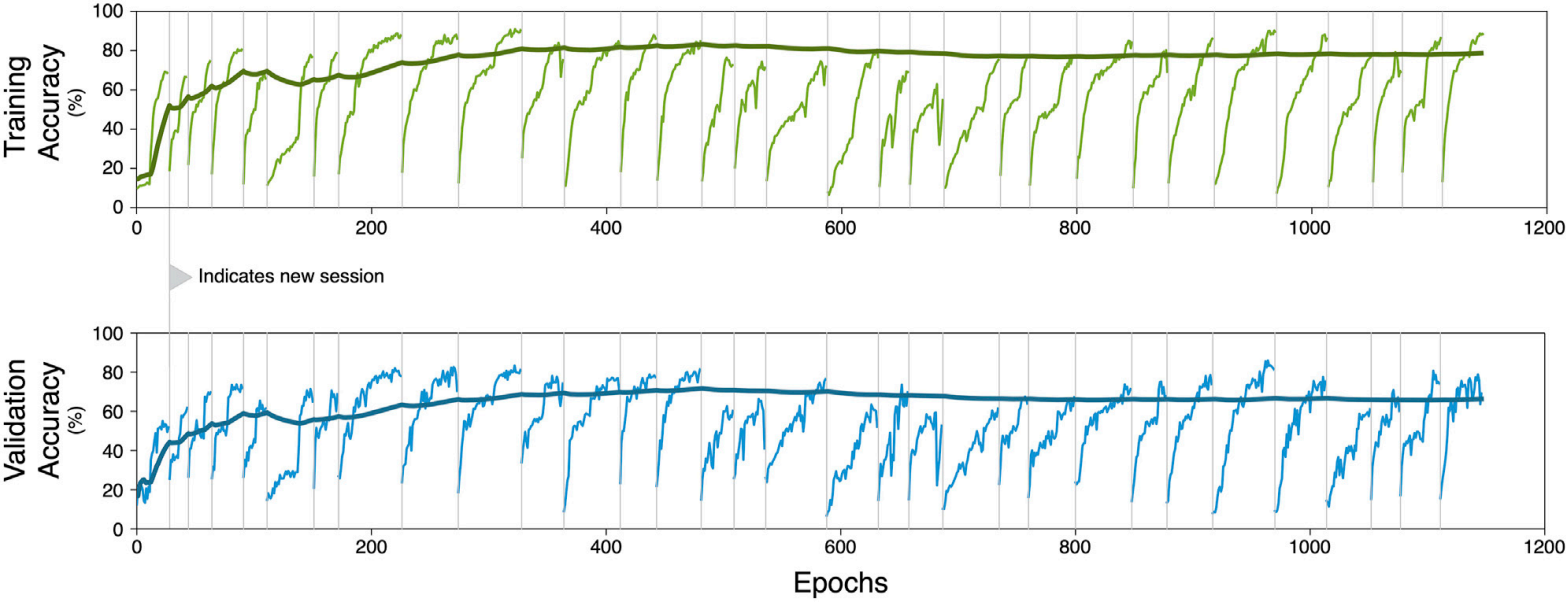
Accuracy during training and validation of the recalibration scheme. Starting with the first training session, performance increases as the number of training epochs increase. Eventually this performance reaches a plateau, at which we begin training on a new session to avoid overfitting on specific sessions. The switch of session data is marked by a vertical line and a subsequent initial drop in performance is observed. The performance accuracy on the overall set of used training data is shown as the bold trendline.

One of the motivations for comparing the three training schemes was to examine any potential benefit of using a large amount of data. As evident by the classification accuracies, it is found that there is benefit to using other sessions during training even if session-specific training must be done. During the recalibration scheme, this beneficial effect starts to plateau at about 14 sessions, with a training accuracy of 80% and a validation accuracy of 72%, suggesting that training on further data provides only small incremental benefits to the encoder-decoder architecture. During recalibration training, we train with a single session at a time because each session has a specific session-calibrator architecture. Whenever a new and unseen session is introduced to train with, the accuracy suffers before rebounding to the plateau. However, the relative impact on the performance of all training data (shown as the trendline in Figure 10), seem to decrease as the number of total epochs and training sessions increase.

## 5 Discussion

The enormous potential for EMG as an interface channel has been recognized for decades. It is a window into the activity of the actuators of the body: muscles. Muscles are the means by which humans interact with the world, beyond generating body poses. They generate forces, stiffness and compliance through antagonist coactivations, with a richness that is not visually apparent. Using EMG we can make devices and systems that seamlessly mesh with those interactions. This motivates the dynamic, temporally diverse gestures we present here. Human interactions are spatiotemporal, transient, and interactive. The utility of a system based on holding a sequence of static poses is limited. Until recently, however, EMG datasets have focused on that case because computational methods have not been adequate for handling dynamic data. Even for static gestures, EMG signals reflect the dynamic physiological activity of muscles, and this complexity amplifies the temporal complexity of the problem. The recurrent neural network method we demonstrate here can learn these complexities.

Practical realizations of a commercial EMG interface are likely to be systems worn daily by the same user. It is not necessary to make a single model for all potential users if we can leverage the data arising from repeated use of a single user. To our knowledge, this dataset is the first to focus on many-session EMG for the same user, and the recalibration scheme is a way to more effectively use that data, providing 79.5% accuracy for the 11 gestures, as opposed to learning solely from the single session (49.6%).

We believe that for a commercially viable XR interaction system or a control scheme for a prosthesis or assistive device, quick and dynamic gestures are more common and natural to users, which is why we believe our dataset to be valuable. We also strive to show the impact of the differences between sessions and how these different sessions can still be used in training an architecture. Therefore, we focus on the increase of performance between the different training schemes.

### 5.1 Architecture choice

The reasoning behind using the encoder-decoder architecture with the bottleneck layer comes from two factors. The first factor is that of visualization of the performance of each architecture. The latent dimensions allows for a low dimensional representation of the data, in which patterns, clusters, and separability is more easily distinguished. While pure performance metrics, like accuracy or RMSE may be adequate to report overall performance, this latent representation affords the opportunity to better understand how an architecture may be learning or representing data. The second factor is that of the potential future uses of this pipeline. Low dimensional representations of high dimensional data can be used in control, demonstrated by Vujaklija et al. (2018), where an autoencoder learned the latent dimension representations of EMG frames and directly extracted these representations for controlling an interface. Similarly, we are developing systems for using low-dimensional EMG control to interface with low-dimensional representations of high-dimensional virtual or prosthetic hands Portnova-Fahreeva et al. (2020, 2022). These applications justify an exploration of using data-driven compression architectures, which when implemented properly, have only a small impact on the performance Dinashi et al. (2022).

### 5.2 Limitations and future work

Differences in data acquisition, number of gestures and subjects, and protocol make it difficult to directly compare these gesture classification results with other similar studies. For example, there are diverse approaches to what EMG frequencies should be included. There is undoubtedly information content in the sub-60 Hz band Sbrollini et al. (2018), but also possible degradation or contamination from other sources, such as motion of the limb, ECG cross-talk, etc. For this reason, most HCI applications use low-frequency cutoffs somewhere between 20Hz and 60Hz López et al. (2018); de Rugy et al. (2012). For this experiment we did not conduct a thorough study of this choice, but chose to remove the low sub-60 Hz frequency content. There is a chance that information from the low frequencies could be used by the learning system to improve performance.

The focus of this study is not to compare classification methods, but to address the problem of generalization across sessions. The second training scheme (Cumulative Training) shows that despite the large number of sessions performed by the same subject, session-specific factors make it difficult to train a network that successfully transfers to a new session. However, by briefly training the session-specific front end calibration network, the data is transformed in such a way that it can be successfully processed by the session-agnostic main network, and can contribute fruitfully to its training. An interesting direction would be to test the effectiveness of the recalibration architecture by passing these transformed features into various other models. Additionally, it would be potentially fruitful to separate the effects of session-specific calibration from the efficacy of the classifier architecture. As we describe in section 2.3.3, other methods have been developed to accommodate inter-session differences. In this manuscript, we do not implement these methods on our dataset for comparison. Architecture design choices, hyperparameter tuning, and similar implementation details can have large effects on the end performance of an approach. To facilitate comparison, we include, alongside the MiSDIREKt dataset, example scripts for loading the dataset and reproducing results presented here. As this research topic matures and more studies are conducted, it will be increasingly important to understand how recalibration methods interact with classification methods and gesture types.

The classification analysis presented in this paper considers a small subset of the dataset, and indeed the most static and isometric poses that were available in the data. The rest of the dataset, which is triple the amount used in this analysis, contains valuable recordings of dynamic movements and interactions with virtual objects. These may hold the key to understanding the relationship between kinematics and EMG, and future works can explore the use of this data for applications like regressive prediction of hand joint kinematics or prosthesis control.

Electrode placement is critical to ensure signal quality, which can degrade through poor surface contact and sliding. The standard placement of the electrode band or array is on the forearm, but recent studies have shown differing effects and quality of data when recording EMG of the wrist Botros et al. (2022). This study found that EMG recordings from electrodes placed on the wrist often provided better quality signals for gestures which involved fine finger movements and had comparable quality to forearm EMG for gestures involving the wrist. These findings translated to the performance of gestures recognition, as classifiers trained on the wrist EMG on average performed better on these fine finger gestures and comparably on the wrist gestures to classifiers trained on the forearm EMG.

As the dataset created and used in this study was for a single subject over many sessions, additional experiments would be required to demonstrate validity for many subjects. Du et al. (2017) and Zhai et al. (2017) have shown the benefit of using retraining on a subject’s multiple sessions. Though these studies use a smaller amount of sessions, these studies use a multitude of subjects, which shows promise for robustness of retraining architectures. With this precedent in mind, future studies which create similar datasets on multiple subjects using the retraining architecture would be a necessary step to show robustness for potential applications.

## 6 Conclusion

This paper presents a dataset, which a novel device design was used to collect, consisting of four distinct tasks, of which some were repeated, of varying dynamic hand movements and tasks: a quick sequence of poses back to back, a sequence of poses where each pose repeated rapidly, a sequence of dynamic interaction gestures held mostly static, and stacking objects to reach a target height repeatedly with different height targets. This dataset collected both EMG and hand kinematic data from hand tracking software within the VR headset. The dataset consists of 43 complete sessions over 12 days, amassing 13.5 h of data, which is one of the largest continuous dynamic datasets publicly available. We perform gesture classification of this dataset using an encoder-decoder architecture for dimensionality reduction. This classification architecture also contains a recalibration process with a session-calibrator architecture to generalize to new sessions, with an accuracy of 79.5%, a marked improvement over the single-session and non-recalibrating architectures which had 49.6% and 55.2% respectively. The success of this architecture on this novel dataset shows promise for more accurate gesture recognition on unseen data, and future works will focus on utilizing the rest of the dataset for dynamic kinematic control through dimensionality reduction architectures.

## Data Availability

Publicly available datasets were analyzed in this study. This data can be found here: https://github.com/maximaka/MiSDIREKt.

## References

[B1] Amma C. Krings T. Böer J. Schultz T. (2015). “Advancing muscle-computer interfaces with high-density electromyography,” in Conference on Human Factors in Computing Systems - Proceedings 2015-April, 929–938. 10.1145/2702123.2702501

[B2] Atzori M. Cognolato M. Müller H. (2016). Deep learning with convolutional neural networks applied to electromyography data: A resource for the classification of movements for prosthetic hands. Front. Neurorobot. 10, 9. 10.3389/fnbot.2016.00009 27656140 PMC5013051

[B3] Atzori M. Gijsberts A. Castellini C. Caputo B. Hager A. G. M. Elsig S. (2014). Electromyography data for non-invasive naturally-controlled robotic hand prostheses. Sci. Data 1, 1–13. 10.1038/sdata.2014.53 PMC4421935 25977804

[B4] Aziz S. Khan M. U. Aamir F. Javid M. A. (2019). “Electromyography (emg) data-driven load classification using empirical mode decomposition and feature analysis,” in Proceedings - 2019 International Conference on Frontiers of Information Technology. 272–277. FIT 2019. 10.1109/FIT47737.2019.00058

[B5] Boe D. Portnova-Fahreeva A. A. Sharma A. Rai V. Sie A. Preechayasomboon P. (2021). Dimensionality reduction of human gait for prosthetic control. Front. Bioeng. Biotechnol. 9, 724626. 10.3389/fbioe.2021.724626 34722477 PMC8552008

[B6] Botros F. S. Phinyomark A. Scheme E. J. (2022). Electromyography-based gesture recognition: Is it time to change focus from the forearm to the wrist? IEEE Trans. Ind. Inf. 18, 174–184. 10.1109/TII.2020.3041618

[B7] Chen X. Zhang X. Zhao Z. Y. Yang J. H. Lantz V. Wang K. Q. (2007). “Multiple hand gesture recognition based on surface emg signal,” in 2007 1st International Conference on Bioinformatics and Biomedical Engineering (New York City, NY: IEEE), 506–509. 10.1109/ICBBE.2007.133

[B8] Cisnal A. Perez-Turiel J. Fraile J. C. Sierra D. Fuente E. D. L. (2021). Robhand: A hand exoskeleton with real-time emg-driven embedded control. Quantifying hand gesture recognition delays for bilateral rehabilitation. IEEE Access 9, 137809–137823. 10.1109/ACCESS.2021.3118281

[B9] Côté-Allard U. Fall C. L. Drouin A. Campeau-Lecours A. Gosselin C. Glette K. (2019). Deep learning for electromyographic hand gesture signal classification using transfer learning. IEEE Trans. Neural Syst. Rehabil. Eng. 27, 760–771. 10.1109/TNSRE.2019.2896269 30714928

[B10] Crawford B. Miller K. Shenoy P. Rao R. (2005). “Real-time classification of electromyographic signals for robotic control,” in Proceedings of the 20th National Conference on Artificial Intelligence - Volume 2 (Palo Alto, CA: AAAI Press), 523–528. AAAI’05

[B11] Cutkosky M. R. (1989). On grasp choice, grasp models, and the design of hands for manufacturing tasks. IEEE Trans. Robot. Autom. 5, 269–279. 10.1109/70.34763

[B12] de Rugy A. Loeb G. E. Carroll T. J. (2012). Virtual biomechanics: A new method for online reconstruction of force from emg recordings. J. neurophysiology 108, 3333–3341. 10.1152/jn.00714.2012 23019006

[B13] Dinashi K. Ameri A. Akhaee M. A. Englehart K. Scheme E. (2022). Compression of emg signals using deep convolutional autoencoders. IEEE J. Biomed. Health Inf. 26, 2888–2897. 10.1109/JBHI.2022.3142034 35015656

[B14] Du Y. Jin W. Wei W. Hu Y. Geng W. (2017). Surface emg-based inter-session gesture recognition enhanced by deep domain adaptation. Sensors (Basel, Switz. 17, 458. 10.3390/S17030458 PMC5375744 28245586

[B15] Fajardo J. M. Gomez O. Prieto F. (2021). Emg hand gesture classification using handcrafted and deep features. Biomed. Signal Process. Control 63, 102210. 10.1016/J.BSPC.2020.102210

[B16] Fang Y. Zhu X. Liu H. (2013). “Development of a surface emg acquisition system with novel electrodes configuration and signal representation,” in Lecture notes in computer science (including subseries lecture notes in artificial intelligence and lecture notes in bioinformatics) 8102 LNAI, 405–414. 10.1007/978-3-642-40852-6_41/COVER

[B17] Feix T. Romero J. Schmiedmayer H.-B. Dollar A. M. Kragic D. (2016). The grasp taxonomy of human grasp types. IEEE Trans. Human-Mach. Syst. 46, 66–77. 10.1109/THMS.2015.2470657

[B18] Hinrichs H. Scholz M. Baum A. K. Kam J. W. Knight R. T. Heinze H. J. (2020). Comparison between a wireless dry electrode eeg system with a conventional wired wet electrode eeg system for clinical applications. Sci. Rep. 10 (10), 1–14. 10.1038/s41598-020-62154-0 32251333 PMC7090045

[B19] Hinton G. E. Salakhutdinov R. R. (2006). Reducing the dimensionality of data with neural networks. Science 313, 504–507. 10.1126/SCIENCE.1127647 16873662

[B20] Ibrahim M. F. I. Al-Jumaily A. A. (2018). Auto-encoder based deep learning for surface electromyography signal processing. Adv. Sci. Technol. Eng. Syst. J. 3, 94–102. 10.25046/AJ030111

[B21] Jabbari M. Khushaba R. N. Nazarpour K. (2020). Emg-based hand gesture classification with long short-term memory deep recurrent neural networks. Annu. Int. Conf. IEEE Eng. Med. Biol. Soc. 2020, 3302–3305. 10.1109/EMBC44109.2020.9175279 33018710

[B22] Jarque-Bou N. J. Vergara M. Sancho-Bru J. L. Roda-Sales A. Gracia-Ibáñez V. (2018). Identification of forearm skin zones with similar muscle activation patterns during activities of daily living. J. NeuroEngineering Rehabil. 15, 91–11. 10.1186/s12984-018-0437-0 PMC6206932 30373606

[B23] Kaczmarek P. Mánkowski T. Tomczýnski J. (2019). putemg—a surface electromyography hand gesture recognition dataset. Sensors (Basel, Switz. 19, 3548. 10.3390/S19163548 PMC6720505 31416251

[B24] Kamakura N. Matsuo M. Ishii H. Mitsuboshi F. Miura Y. (1980). Patterns of static prehension in normal hands. Am. J. Occup. Ther. 34, 437–445. 10.5014/AJOT.34.7.437 6446851

[B25] Kim J. Mastnik S. André E. (2008. “EMG-based hand gesture recognition for realtime biosignal interfacing,” in Proc. 13th Int. Conf. Intell. User Interfaces, 30–39.

[B26] Kong H. Lu L. Yu J. Chen Y. Tang F. (2021). Continuous authentication through finger gesture interaction for smart homes using wifi. IEEE Trans. Mob. Comput. 20, 3148–3162. 10.1109/TMC.2020.2994955

[B27] Li S. Liu C. H. Lin Q. Wen Q. Su L. Huang G. (2021). Deep residual correction network for partial domain adaptation. IEEE Trans. Pattern Anal. Mach. Intell. 43, 2329–2344. 10.1109/TPAMI.2020.2964173 31944945

[B28] López D. A. R. Correa H. L. López M. A. Sánchez J. E. D. (2018). Expert committee classifier for hand motions recognition from emg signals. Ingeniare. Rev. Chil. Ing. 26, 62–71. 10.4067/s0718-33052018000100062

[B29] Luca C. J. D. Gilmore L. D. Kuznetsov M. Roy S. H. (2010). Filtering the surface emg signal: Movement artifact and baseline noise contamination. J. Biomechanics 43, 1573–1579. 10.1016/J.JBIOMECH.2010.01.027 20206934

[B30] Ma X. Rizzoglio F. Perreault E. J. Miller L. E. Kennedy A. (2022). Using adversarial networks to extend brain computer interface decoding accuracy over time. bioRxiv, 2022.08.26.504777. 10.1101/2022.08.26.504777 PMC10446822 37610305

[B31] Matsugi A. Yoshida N. Okada Y. Marcos-Antón S. Gor-García-Fogeda M. D. de-la Cuerda R. C. (2022). An semg-controlled forearm bracelet for assessing and training manual dexterity in rehabilitation: A systematic review. J. Clin. Med. 2022 11, 3119. 10.3390/JCM11113119 PMC9181798 35683503

[B32] Portnova-Fahreeva A. A. Rizzoglio F. Casadio M. Mussa-Ivaldi F. A. Rombokas E. (2022). “Learning to operate a high-dimensional hand via a low-dimensional controller,” in Review. 10.3389/fbioe.2023.1139405 PMC10192906 37214310

[B33] Portnova-Fahreeva A. A. Rizzoglio F. Nisky I. Casadio M. Mussa-Ivaldi F. A. Rombokas E. (2020). Linear and non-linear dimensionality-reduction techniques on full hand kinematics. Front. Bioeng. Biotechnol. 8, 429. 10.3389/fbioe.2020.00429 32432105 PMC7214755

[B34] Prechayasomboon P. Rombokas E. (2023). “Actually active electrodes: Pneumatically actuated electrodes for emg-based interaction in virtual reality,” in Review.

[B35] Preechayasomboon P. Rombokas E. (2021). Haplets: Finger-worn wireless and low-encumbrance vibrotactile haptic feedback for virtual and augmented reality. Front. Virtual Real. 2, 1–15. 10.3389/FRVIR.2021.738613

[B36] Resnik L. J. Acluche F. Klinger S. L. (2018). User experience of controlling the deka arm with emg pattern recognition. PLOS ONE 13, e0203987. 10.1371/JOURNAL.PONE.0203987 30240420 PMC6150511

[B37] Sbrollini A. Strazza A. Candelaresi S. Marcantoni I. Morettini M. Fioretti S. (2018). Surface electromyography low-frequency content: Assessment in isometric conditions after electrocardiogram cancellation by the segmented-beat modulation method. Inf. Med. Unlocked 13, 71–80. 10.1016/j.imu.2018.10.006

[B38] Schmid P. J. (2010). Dynamic mode decomposition of numerical and experimental data. J. Fluid Mech. 656, 5–28. 10.1017/S0022112010001217

[B39] Sebelius F. C. Rosén B. N. Lundborg G. N. (2005). Refined myoelectric control in below-elbow amputees using artificial neural networks and a data glove. J. Hand Surg. 30, 780–789. 10.1016/J.JHSA.2005.01.002 16039372

[B40] Siami-Namini S. Tavakoli N. Namin A. S. (2019). “The performance of lstm and bilstm in forecasting time series,” in Proceedings - 2019 IEEE International Conference on Big Data, Big Data 2019, 3285–3292. 10.1109/BIGDATA47090.2019.9005997

[B41] Simão M. Neto P. Gibaru O. (2019). Emg-based online classification of gestures with recurrent neural networks. Pattern Recognit. Lett. 128, 45–51. 10.1016/J.PATREC.2019.07.021

[B42] Simão M. Neto P. Gibaru O. (2018). Uc2018 dualmyo hand gesture dataset. 10.5281/ZENODO.1320922

[B43] Spiewak C. (2018). A comprehensive study on emg feature extraction and classifiers. Open Access J. Biomed. Eng. Biosci. 1. 10.32474/OAJBEB.2018.01.000104

[B44] Vujaklija I. Shalchyan V. Kamavuako E. N. Jiang N. Marateb H. R. Farina D. (2018). Online mapping of emg signals into kinematics by autoencoding. J. NeuroEngineering Rehabil. 15, 21–29. 10.1186/s12984-018-0363-1 PMC5850983 29534764

[B45] Wen R. Nguyen B. P. Chng C. B. Chui C. K. (2013). “ *In situ* spatial ar surgical planning using projector-kinect system,” in ACM International Conference Proceeding Series, 164–171. 10.1145/2542050.2542060

[B46] Wen R. Tay W. L. Nguyen B. P. Chng C. B. Chui C. K. (2014). Hand gesture guided robot-assisted surgery based on a direct augmented reality interface. Comput. methods programs Biomed. 116, 68–80. 10.1016/J.CMPB.2013.12.018 24438993

[B47] Xie B. Li S. Lv F. Liu C. H. Wang G. Wu D. (2022). A collaborative alignment framework of transferable knowledge extraction for unsupervised domain adaptation. IEEE Trans. Knowl. Data Eng, 9803869. 10.1109/TKDE.2022.3185233

[B48] Zhai X. Jelfs B. Chan R. H. Tin C. (2017). Self-recalibrating surface emg pattern recognition for neuroprosthesis control based on convolutional neural network. Front. Neurosci. 11, 379. 10.3389/fnins.2017.00379 28744189 PMC5504564

[B49] Zhang Z. He C. Yang K. (2020). A novel surface electromyographic signal-based hand gesture prediction using a recurrent neural network. Sensors 2020 20, 3994. 10.3390/S20143994 PMC7412393 32709164

[B50] Zheng N. Li Y. Zhang W. Du M. (2022). User-independent emg gesture recognition method based on adaptive learning. Front. Neurosci. 16, 847180. 10.3389/fnins.2022.847180 35431778 PMC9008251

